# Epidemiology and Histopathology of Nasopharyngeal Neoplasms in Iran

**DOI:** 10.22038/IJORL.2022.63193.3180

**Published:** 2023-01

**Authors:** Ali Safavi Naini, Mohammad Mehdi Rostami, Fatemeh Shojaeian, Mehdi Azizmohammad Looha, Ali Ghanbari Motlagh, Amir Ali Safavi, Seyed Amir Ahmad Safavi-Naini

**Affiliations:** 1 *National Research Institute of Tuberculosis and Lung Diseases, Massih Daneshvari Hospital, Shahid Beheshti University of Medical Sciences, Tehran, Iran.*; 2 *Sidney Kimmel Comprehensive Cancer Center, Johns Hopkins University School of Medicine, Baltimore, Maryland, US.*; 3 *Basic and Molecular Epidemiology of Gastrointestinal Disorders Research Center, Research Institute for Gastroenterology and Liver Diseases, Shahid Beheshti University of Medical Sciences, Tehran, Iran.*; 4 *Cancer Office, Deputy of Health, Ministry of Health, Tehran, Iran.*

**Keywords:** Nasopharyngeal Neoplasms, Nasopharyngeal Carcinoma, Epidemiology, Histology, Incidence, Iran

## Abstract

**Introduction:**

This study aimed to study the trend, histologic pattern, geographical distribution, and characteristics of nasopharyngeal carcinoma (NPC) and nasopharyngeal neoplasms (NPN) from 2003 to 2017 in Iran.

**Materials and Methods:**

The Ministry of Health and Medical Education collected NPN cases from the corresponding university in each province and stored them in Iran National Cancer Registry (INCR) database. The Joinpoint program calculated the average annual percent change (AAPC) and its 95% confidence interval (CI). The jump model minimized the interfering effect of INCR transformation.

**Results:**

3653 NPN cases were reported between 2003-2010 and 2014-2017, with a mean age of 49.04 ± 18.31 years and a male-to-female ratio of 2.15. The age-standardized incidence rate (ASIR) per 100,000 person-years was 0.30 for females and 0.68 for males in 2017. Although the ASIR/100,000 of NPN raised from 0.35 to 0.49 during 2003-2017, the trend was constant with an AAPC of -2% (95% CI: -4.8% to 0.9%). The age-specific incidence rate was highest in the older than 70 population (1.56/100,000). NPC formed 77.1% of NPNs and showed a constant pattern (AAPC CI: -5.7% to 0.2%), in contrast to the significant increase of non-keratinizing squamous cell carcinoma (AAPC CI: 2.3%to 24.5%).

**Conclusions:**

Nasopharynx cancer is rare in Iran, and NPC incidence remained constant from 2003 to 2017, unlike previously reported rising trend. However, non-keratinizing squamous cell carcinoma exhibited a significant increase, and future studies are needed to examine the role of the Epstein-Barr virus on this growth rate.

## Introduction

Different Nasopharyngeal Neoplasms (NPNs) can appear in the nasopharynx, such as carcinoma, hematolymphoid, and soft tissue tumors, which arise from epithelial, mesenchymal, lymphoid, and neuroectodermal tissues (1). 

NPN can be caused by different risk factors, including genetic predisposition, smoking, alcohol consumption, salted fish intake, and the Epstein-Barr virus (EBV) (2–5). However, late clinical presentation, lack of knowledge of molecular mechanisms, and poor treatment response made Nasopharyngeal Carcinoma (NPC) a malignancy with high mortality (6). 

Nonetheless, there are some controversies in the pathological classification of NPC (7,8). The relationship with the EBV, radiotherapy sensitivity, and a higher rate of distant metastasis made NPC different from other non-nasopharyngeal head and neck squamous cell carcinomas (9). 

The male population has a higher risk of NPC, possibly due to the higher related risk factors, mainly smoking, alcohol consumption, and workplace exposure (10).

NPC is the most common malignancy in the nasopharynx, often diagnosed with poorly or undifferentiated carcinoma (9,11,12). More than 100 thousand cases are diagnosed with NPC cancer yearly (13). 

Besides, the NPC incidence ranges from 1 per 100,000 person-years, in most of the world, to 20 per 100,000 person-years in endemic countries (13). 

Despite the low prevalence of NPC in the Western area, endemic countries, such as southern China, Southeast Asia, north Africa, and the Arctic, face high mortality rates and incidence rates (2,3,10,14). This diverse incidence means NPC accounts for 2% to 60% of all head and neck cancers (15,16). 

As NPC has variable incidence rates worldwide and is associated with genetic, ethnicity, and geographical features, it seems essential for each country to investigate the epidemiological characteristics of this malignancy. Our previous studies found an incidence rate of 0.38 per 100,000 person-years over six years, with an increasing trend (17,18). This study aimed to investigate the trend, geographical distribution, and characteristics of NPN from 2003 to 2017 in Iran. 

## Materials and Methods

*Data source and population*

The institutional review board approved the study, and the confidentiality of patients' information was a concern during the analysis. Data on unduplicated cases of nasopharyngeal cancer during 2003-2010 and 2014-2017 was collected from Iran's National Cancer Registry (INCR). Due to administrative issues, no data was available in 2011, 2012, and 2013. In these three years, the cancer statistics for the nasopharynx had extensively low coverage and faced underreporting. Therefore, the INCR avoids providing the data for the investigation to prevent incorrect results.

A new cancer registry was established in 2014 to achieve population-based registry standards; therefore, cancer data was gathered from pathology reports, clinical diagnoses, and death certificates between 2014 and 2017, while it was only based on pathology reports between 2003 and 2010. The INCR program is national, has mandatory registration, and is grounded in the Ministry of Health and Medical Education (MOHME) cancer office. Each region has a corresponding medical university responsible for gathering the cancer statistics and registration of the subjects’ data in a national registration software (Sima-E-saratan). MOHME's cancer office collected cancer data from all universities. All health centers, including hospitals, clinics, and pathology laboratories, were required to report patient information to the MOHME. 

More information on population-based INCR is available in INCR guidelines and instructions (19).Age, gender, date of birth, method of diagnosis (pathologic, clinical, death certificate only), date of diagnosis, morphology code, topography code, grade of tumor, and province of residence were defined in the data set for each patient. We defined a 5-year increment age group (e.g., 0-4, 5-9… 80-84, >85) for cases. 

*Data quality and processing*

In some years, there were problems with data, such as multiple recorded data for some patients. Incorrect morphology may have been recorded for a topography associated with nasopharyngeal cancer. Furthermore, the diagnosis of nasopharyngeal cancer could be incorrect or invalid. Moreover, the patients' birthdates and ages differed in some cases. As a result, the data quality needs to be assessed. The cancer office of MOHME handled duplicated cases before using the patients' IDs and the cases' personal information. In addition, consistency was assessed by analyzing the topography and morphology of patients. A re-examination was conducted if there was any inconsistency, and the subject would be excluded if the data were inaccurate.The type of tumor was assessed afterward to determine whether it was diagnosed as nasopharyngeal cancer. Following this, the accuracy of patients' birthdays and the date of their cancer diagnosis was checked; any inaccurate information was corrected or removed from the dataset. A cancer diagnosis was determined based solely on the pathology (or cytology), clinical outcome, and death certificate. Data completeness for the sex, age, morphology code, and province variables are presented in Supplementary 1. By using a capture-recapture method, Mohammadi et al. evaluated the internal completeness of the age, sex, and morphology code during 2008-2010 and concluded that the completeness of INCR was 96.4% (20).

*Nasopharyngeal cancer classification*

Based on the international classification of diseases for oncology (ICD‑O: Topography, ICD‑OM: Morphology), a topography code was reported for nasopharyngeal cancer (C11), with the location of malignancy codes defined as the superior wall (C11.0), posterior wall (C11.1), lateral wall (C11.2), the anterior wall of the nasopharynx (C11.3), and not otherwise specified (NOS) (C11.9). ICD-O morphology codes were used to determine histology types. NPN cancers were then presented as major groups of NPCs, hematolymphoid malignancies, other nasopharyngeal cancers, and unclassified cancers. There are different NPC classifications, but in this study, NPC was classified as carcinoma NOS, undifferentiated carcinoma, squamous cell carcinoma (SCC) NOS, keratinizing SCC (K-SCC), and non-keratinizing SCC (NK-SCC). This form of reporting makes it possible to align the results of this study with the Chinese or World Health Organization (WHO) classification.

*Statistical analysis*

The Joinpoint regression program 4.8.0.1 (Statistical Research and Applications Branch, National Cancer Institute) was used to calculate annual percent change (APC), average annual percent change (AAPC), the 95% confidence interval of APC, and joinpoint regression analysis. The Joinpoint regression, the weight least-square method, and the weighted Bayesian Information Criteria (BIC). 

The minimum and the maximum number of joinpoints were set as zero and two, respectively. We also used the advanced jump model of the program from 2010 to 2014 (due to the lack of information in 2011-2013 and the transformation to the population-based cancer registry system from the pathologic-based cancer registry system). Therefore, the level shift in ASIR due to higher cancer detection has a less interfering effect on the trend of ASIR, and the Joinpoint-Comparability Ratio was calculated (21). The joinpoint analysis was performed for male, female, and morphology groups from 2003 to 2017. Since there was a significant missing value from 2003-2006 in local data, we studied the trend in each province from 2007 to 2017 to calculate AAPC. The Bonferroni correction was used for multiple tests in the program. The Monte Carlo permutation test was used to find the best-fitted trendline to determine the significance of trends. Changes in the trend were statistically significant at a significance level of 0.05. The NPN data of INCR was unavailable during 2010-2012; hence, we used a prediction model to examine and illustrate the time trend. 

The 3-year-ahead projection of cancer incidence was estimated using Holt’s linear, exponential smoothing method based on the ASIR data from 2003 to 2010 among females and males. This method is suitable for small sample data (22). The 95% CI and the 2011 predicted value illustrated the trend line. The incidence rate per 100,000 person-years, ASIR per 100,000 person-years, and the age-specific incidence rate per 100,000 person-years were estimated by age-group populations according to the Statistical Centre of Iran Census (SCIC). Data for the male and female population in 2016, 2011, and 2006 was available by SCIC, and the population of other years was estimated through a growth rate between two consecutive censuses. According to the WHO's World Standard Population ASIR was calculated (23). The continuous and categorical variables are summarized as mean ± standard deviation and numbers or percent. The Kolmogorov-Smirnov test confirmed the normal age distribution. The p-values less than 0.05 were regarded as statistically significant. All analysis was performed by the R (4.0.2) program. 

## Results

A total of 3653 new NPN cases were registered in the national cancer registry database between 2003 and 2010 and 2014-2017. The mean age of the patients was 49.04 ± 18.31 years, and the male-to-female ratio was 2.15. Microscopically, 79.8% of cases were verified between 2014 and 2017, while 15.2% and 5% were documented by clinical findings and death certificates, respectively. Seventy-six deaths were reported with a mean age of 64.68 ± 19.50, and the male-to-female ratio was 2.4. The mortality-to-incidence ratio for nasopharyngeal neoplasms was 0.02 from 2014 to 2017. 

*The nasopharyngeal neoplasm incidence and trend*

**Fig 1:**
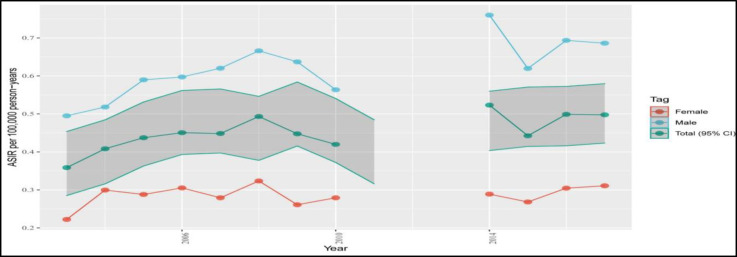
The age-standardized incidence rate per 100,000 person-years trends of nasopharyngeal cancer during 2003-2017 among males, females, and total patients (CI: confidence interval).

The ASIR of NPN in 2017 was 0.68 and 0.30 per 100,000 person-years among males and females, respectively (Figure 1). Although the ASIR/100,000 of NPN raised from 0.35 to 0.49 during 2003-2017, the overall trend remained constant with an AAPC of -2% (-4.8%-0.9%). The male and female incidence rate of NPN was also constant during the study period (Table 1). 

Supplementary 2 illustrates the joinpoint analysis of the trend, considering INCR transformation from pathologic-based to population-based. Accordingly, the Joinpoint-comparability ratio from 2010 (pathologic-based INCR) to 2014 (population-based INCR) was 1.79 (95% CI: 0.02-3.55).

**Table 1:** The trend analysis and average annual percent change for histology groups of nasopharyngeal cancers, males and females, and total patients during 2003-2017 using the jump model from 2010 to 2014

	**2003-2017**		**Trend 1**			**Trend 2**			**Trend 3**		
	**AAPC (95% CI)**	**P-value**	**Year**	**AAPC (95% CI)**	**P-value**	**Year**	**(95% CI)**	**P-value**	**Year**	**AAPC (95% CI)**	**P-value**
Total	-2 (-4.8 - 0.9)	0.17	2003-2008	5.7* (0.7 - 11)	0.03	2008-2015	-8.9* (-12.6 - -5.1)	0.001	2015-2017	4.5 (-15.9 - 29.9)	0.6
Male	-2.3 (-5.7-1.2)	0.2	2003-2008	6.6* (0.5-13.1)	0.04	2008-2015	-9.5* (-13.9--4.8)	0.01	2015-2017	2.8 (-21-33.9)	0.78
Female	1.3 (-3.6-6.4)	0.62	2003-2005	14.6 (-17-58.3)	0.31	2005-2015	-2.2 (-5.7-1.4)	0.16	2015-2017	6.7 (-22.8-47.3)	0.61
NPC	-2.8 (-5.7-0.2)	0.07	2003-2008	5.4* (0.2-11)	0.05	2008-2015	-10.0* (-13.8--6)	0	2015-2017	4 (-17.3-30.7)	0.66
Undifferentiated carcinoma	-4.4 (-9.8-1.2)	0.12	2003-2008	7 (-2.9-17.9)	0.12	2008-2015	-13.6* (-20.4--6.2)	0.01	2015-2017	2.4 (-33.6-57.8)	0.89
SCC	-0.8 (-3.3-1.7)	0.47									
Non-keratinizing SCC	12.8* (2.3-24.5)	0.02	2003-2010	-2.7 (-11.3-6.7)	0.5	2010-2017	30.9* (5.2-63)	0.02			
Keratinizing SCC	-9.1 (-36.2-29.6)	0.56									
Adenocarcinoma	-0.3 (-4.1-3.6)	0.86									
Hematolymphoid	0.4 (-9.7-11.5)	0.95	2003-2006	28.4 (-12-87.4)	0.14	2006-2015	-10.5* (-18.9--1.1)	0.04	2015-2017	15.9 (-45.6-146.8)	0.62

Footnote: NPC: nasopharyngeal carcinoma, SCC: squamous cell carcinoma, AAPC: average annual percent change.

*The age-specific* *incidence* *rate of nasopharyngeal neoplasm*

**Fig 2:**
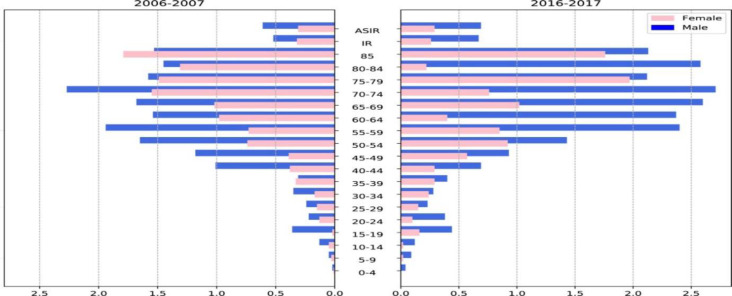
The age-specific incidence rate (IR) and age-standardized incidence rate (ASIR) of nasopharyngeal cancer per 100,000 people in 2006-2007 and 2016-2017

As shown in Figure 2, the age-specific incidence rate increased with age. The risk of NPN before 45 years old was low, with a value of 0.19/100,000. Incidence peaked at 1.30/100 

 and 1.56/100,000, respectively, for the 45-70 and older 70 age groups. Both sexes followed this pattern, but men's NPN peak was more pronounced. There was also a slight increase in NPN incidence among the population aged 15-25, but it was not statistically significant.

*The trend of histologic types of NPN*

Table 2 shows the histologic characteristics of NPN. NPC (77.1%) was the most common type of NPN, consisting of carcinoma NOS (14.6%), undifferentiated carcinoma (44.6%), SCC carcinoma (17.8%), Non-keratinizing SCC (7.3%), and keratinizing SCC (1%). Male-to-female ASIR ratios were lowest for hematolymphoid neoplasms and keratinizing SCC (1.67); however, the highest ratio was observed for undifferentiated carcinoma (3.19). As shown in Table 1, all histology groups showed a constant trend, except for non-keratinizing SCC, which had a rising trend in 2017 (Supplementary 2). Adenocarcinoma (N=66), rhabdomyosarcoma (N=25, average age ± SD = 14.84 ± 13.69, M: F ratio=1.6), and mucoepidermoid neoplasms (N=8, average age ± SD = 53.87 ± 19.17, M: F ratio=1) were among the rare NPNs (Supplementary 3)

**Table 2:** The demographics, age standard incidence rate per 100,000 person-years, average annual percent change, and trend of nasopharyngeal neoplasms from 2003 to 2017

	**Cases (percent among all cases)**	**Male ASIR 2016-2017**	**Female ASIR 2016-2017**	**M: F ASIR ratio**	**Average age±SD**	**2003 ASIR**	**2017 ASIR**	**AAPC**	**Trend of ASIR in 2017**
Total	3653 (100%)	0.689	0.307	2.24	49.04±18.31	0.359	0.498	-2 (-4.8 - 0.9)	Constant
1. NPC	2816 (77.1%)	0.492	0.183	2.69	48.66±17.48	0.309	0.342	-2.8 (-5.7-0.2)	Constant
1.2. Undifferentiated Carcinoma	1629 (44.6%)	0.252	0.079	3.19	46.63±17.5	0.174	0.172	-4.4 (-9.8-1.2)	Constant
1.3. SCC Carcinoma	651 (17.8%)	0.122	0.053	2.3	53.79±16.47	0.094	0.092	-4.4 (-9.8-1.2)	Constant
1.3.1 Non-keratinizing SCC	268 (7.3%)	0.065	0.019	3.42	49.51±17.84	0.05	0.044	12.8* (2.3-24.5)	Rising
1.3.2. Keratinizing SCC	34 (0.9%)	0.005	0.003	1.67	58.06±13.72	0.008	0.002	-9.1 (-36.2-29.6)	Constant
2. Adenocarcinoma	66 (1.8%)	0.01	0.009	1.11	51.37±15.28	0.008	0.009	-0.3 (-4.1-3.6)	Constant
3. Hematolymphoid	321 (8.8%)	0.053	0.031	1.71	50.17±20.28	0.024	0.049	0.4 (-9.7-11.5)	Constant

Footnote: SD: standard deviation, M:F: male to female ratio, SCC: squamous cell carcinoma, NOS: not otherwise specified. AAPC: average annual percent change, ASIR: age standard incidence rate

*Neoplasm site and grading of nasopharyngeal neoplasms*

In total, 96.7% of tumor sites were not otherwise specified. Among the 108 defined cancer sites, the posterior wall of the nasopharynx (45.5%) was the most common cancer site, followed by the anterior wall (23.1%), lateral wall (19.8%), and superior wall (11.5%) (Supplementary 4). A total of 1889 cases had a report of pathologic grade. Most of NPNs were undifferenced (80.56%) or poorly differentiated (11.76%) (Supplementary 5).

*Trend and incidence of nasopharyngeal*

*neoplasm in 31 provinces of Iran*

**Fig 3:**
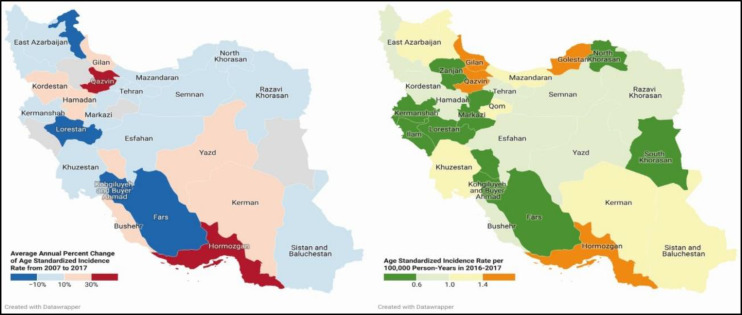
The density map of age-standardized incidence rate per 100,000 person-years in 2016-2017 and percent change from 2007 to 2017 using jump model of joinpoint analysis for nasopharyngeal neoplasms

 Figure 3 illustrates the ASIR during 2016-2017 and the AAPC during 2007-2017 for 31 provinces of Iran. ASIR rose statistically significantly in eight provinces (AAPC: Hormozgan: 52.4%, Qazvin: 37.3%, Gilan: 29.5%, Kordestan: 28.2%, Bushehr: 23.7%, Kerman: 15.1%, Kerman: 15.1%, Hamedan: 11.6%). Furthermore, ten provinces declined (Lorestan, Kohgiluyeh and Buyer Ahmad, Fars, Ardebil, Kermanshah, Markazi, Esfahan, Mazandaran, Khuzestan, Razavi-Khorasan), and the other nine provinces had a constant trend. Supplementary 6 provides each province’s ASIR and AAPC (using an advanced jump model from 2010 to 2014).

## Discussion

The current study was the first to describe the trend of nasopharyngeal neoplasm using demographic and histologic characteristics in the last decade in Iran. 

Each year, about one individual per 300,000 women and 150,000 men is diagnosed with nasopharyngeal cancer in Iran. Our study showed that the previous rising trend of NPN in Iran (17) has became constant in recent years.

NPC incidence was stable, in contrast to the rising rate of non-keratinizing carcinomas (NK-NPC), which might be related to the EBV infection. In line with GLOBOCAN 2020, NPN is a rare disease with a lower incidence in Iran than in other countries, including Arab/non-Arab neighbors (24). A joinpoint comparability ratio of 1.79 indicates that the transformation of INCR to the population-based registry system increased the detection rate of NPNs.

One of NPC's epidemiological characteristics is that its incidence and mortality vary by histology, geography, gender, and age (2,25). The Middle East is considered a region with an average NPC incidence rate of 2.7 (24). Countries with moderate and high human development index (HDI) show a higher risk of NPN. Aligned with previous studies, we found a lower incidence rate of nasopharyngeal cancer in Iran than in other countries with high HDI or located in the middle east and north African region (26–28). Moreover, the global ASIR/100,000 decreased from 1.88 in 1990 to 1.35 in 2017. Therefore, the APC of incidence we found in Iran (-2%) is comparable to the global incidence (APC~ -1%). Also, the trend of unadjusted incidence in Iran seems to parallel the global trend (APC~ 1%) (13). The low incidence of NPC in Iran might be explained by Iranian lifestyle, genetic factors, or inefficiency of the registry, which will be discussed in the following.

Based on rare previous surveys, a low EBV infection rate was evident among blood donors and gastric cancer cases (29,30). Almost all patients with hematologic malignancies tested positive for EBV in Saudi Arabia, while they were positive in only 46% of patients in Iran (31,32). Also, NPC occurs a couple of years after EBV infection and is more related to NK-NPC (33). Therefore, a low EBV infection rate in the old population may explain some extent of rare NPC incidence in Iran. However, the older generation of Iran's population had healthier sexual behavior, unlike younger people (34). A study showing 90% EBV-positives among young adults in Tehran may confirm the latter (35). In our study, the rise of NK-NPC, a malignancy more related to EBV, may warn that the constant trend of NPC may change over time. Also, The other routes of EBV transmission should be considered, such as through the mother’s saliva to the child (36). The mentioned hypothesis is based on limited literature and the results of this observational study. EBV is necessary but insufficient for oncogenesis, and multiple factors are involved in NPC oncogenesis (37). Hence, further studies are highly recommended to evaluate the EBV incidence rate among Iranian NPC patients. Genetic susceptibility and ethnicity may elucidate the heterogeneous pattern of NPC incidence. A high NPC incidence was observed among Southeast Asians and Northern Africans. Iran is a mixture of Persian, Turk, Kurd, and Arab ethnic groups, mainly Indo-Europeans (38). Kuwaitis are similar to Iranians in ethnicity and have a low NPC incidence rate (36). In contrast, other counties, such as Afghanistan, Tajikistan, and Azerbaijan, have a slightly higher NPC incidence rate (24). Based on Patel et al. study on NPC survival rates among different racial groups (39), there was a disadvantage of Caucasian ethnicity in the prognosis of nasopharynx cancer. However, "Caucasian" is used frequently to describe ethnicity in the literature, while it refers to a heterogeneous group and not a specific race (40). However, more detailed studies are needed to assess the effect of Persian ethnicity on NPC. 

In low-risk countries, there was a bimodal age pattern with two peaks in age groups same as in Iran. The first minor peak was in early adulthood (15-24 years old), and the second significant peak was in patients aged 65-79 years. Contrastively, in the high-risk population, a unimodal age pattern was noted, with the incidence peaking only in patients aged 65-79 (25). In Chinese people, the risk rises dramatically above 30 years of age and increases until 45-54 years of age, then declines (43). The current study found that the peak incidence rate was among males 65-69 and females over 75. We also detected a non-significant peak in early adulthood (15-25), similar to other low-risk countries (25). Rhabdomyosarcomas shapes 50 percent of NPN in the 0-10 age group. A minor peak in undifferentiated carcinoma (15-25 age group) and lymphoma (10-20 age group) makes a slight peak in the total ASIR of patients aged 15-25 years, which has become more significant in recent years (Figure 2). Iran showed a bimodal pattern with a minor peak in patients aged 15-25 years and a significant peak in patients aged 70 years, consistent with other low-risk countries. The early adulthood peak suggests the role of genetic susceptibility, and the late-adolescence peak is more related to exposure to NPC risk factors (25). In our study, the male gender had a higher ASIR with a male-to-female ratio of 2.15, consistent with most similar studies in other countries (14). A recent study showed that higher vascular endothelial growth factor polymorphism in the male gender could explain the volubility of males (44).

NPC is the most common type of nasopharyngeal cancer. The NPC classification has always been controversial, and WHO, China, the union for international cancer control, and different scientists and physicians worldwide have proposed a variety of NPC classifications (8). Undifferentiated carcinoma is the most frequent histological subtype of NPC, consistent with our study, and highly correlated with EBV. The increasing number of patients with undifferentiated carcinoma might suggest growth in EBV prevalence in the Iranian population, which is in line with the change in sexual behavior among young generations (34). However, differentiated carcinoma constitutes a significant part of NPC, up to about 25% (45). K-NPC is extremely rare in Iran, and trend analysis was not achievable, while the incidence seems constant (Supplementary 2). In another study in Hong Kong, the incidence rate of keratinizing carcinoma decreased associated with reducing cigarette smoking (46). It has been shown that NK-SCC and K-SCC had the best and worst prognoses, respectively (47). NK-NPC was even more frequent than K-NPC among males and older patients (Table 2); this was also evident in other studies (48). The rhabdomyosarcoma of NPC mainly occurred in patients aged 0-10 years, which is consistent with other studies (49). Also, there was a minor peak of undifferentiated carcinoma and hematolymphoid malignancies in early adulthood. EBV has a possible role in both malignancies, while the pathway at this young age is not well understood (49). In line with previous studies, nasopharyngeal lymphomas accounted for 10 percent of NPNs, and large B-cell lymphoma was the most common subtype (37). Currently, initial misdiagnosis is common and takes about six months from the onset of symptoms to treatment, even with endoscopic tools. Understanding risk factors associated with NPC can help determine higher risk groups and detect cancer early. EBV, smoking, alcohol intake, male gender, and age >54 years were NPC risk factors. (50). Early-stage detection is the most important prognostic factor of head and neck cancers; it can also be beneficial (51). There are some challenges to upcoming EBV vaccines; until then, hygiene improvement may help prevent the infection (52). Dietary modification and sexual health education may be effective interventions in Iran. As Figure 3 depicts, Guilan and Hormozgan, two provinces with the highest NPC incidence, are known provinces with higher fish consumption. They can be a good focus for implementing prevention programs.

There are many limitations to this study. We provided possible explanations only based on observational studies. Neither lifestyle and environmental risk factors nor the size and staging of the tumor were available. Also, cancer location and grading data were incomplete, and there were other external incompleteness and validity issues with Iran's cancer database. Although efforts to conduct a population-based registry system increased the detection rate, it takes time to establish a good registry system. However, we attempted to minimize the interfering effect of higher detection rates on the trend of NPN incidence, using prediction models and the jump model of the Joinpoint program.

## Conclusion

Iran is a low-risk country for nasopharyngeal neoplasms, and each year, one individual per 200,000 people is diagnosed with this cancer. Men were twice as likely to be involved with NPN than women, and a bimodal age pattern was observed in Iran with a minor peak of incidence in the 10-20 age group and a significant incidence in old ages. During 2003-2017, nasopharynx cancer and NPC were constant, contrary to the rising trend reported previously. However, non-keratinizing SCC had a significant rising, and further studies are highly recommended to assess the role of EBV in this growth rate. Hormozgan, Gilan, and Qazvin provinces are priorities for establishing prevention programs. 

## Conflict of Interests

The author (A.M.) is a deputy of the cancer prevention office in the health and medical education ministry. The authors declare no other conflict of interest related to this work.

### Data Availability Statement

The results and dataset can be available per reasonable request to the corresponding author and under the permission of ministry of health and medical education. However, the ASIR in 31 provinces of Iran is available in Supplementary File 6 for further study of NPC risk factors in Iran. 
